# Early-Life Socioeconomic Position and the Accumulation of Health-Related Deficits by Midlife in the 1958 British Birth Cohort Study

**DOI:** 10.1093/aje/kwab038

**Published:** 2021-02-17

**Authors:** Nina T Rogers, Joanna M Blodgett, Samuel D Searle, Rachel Cooper, Daniel H J Davis, Snehal M Pinto Pereira

**Keywords:** birth cohort, childhood circumstances, early-life socioeconomic position, frailty, healthy aging, life course, socioeconomic status

## Abstract

Reducing population levels of frailty is an important goal, and preventing its development in midadulthood could be pivotal. There is limited evidence on associations between childhood socioeconomic position (SEP) and frailty. Using data on the 1958 British birth cohort (followed from 1958 to 2016; *n* = 8,711), we aimed to 1) establish the utility of measuring frailty in midlife, by examining associations between a 34-item frailty index at age 50 years (FI_50y_) and mortality at ages 50–58 years, and 2) examine associations between early-life SEP and FI_50y_ and investigate whether these associations were explained by adult SEP. Hazard ratios for mortality increased with increasing frailty; for example, the sex-adjusted hazard ratio for the highest quintile of FI_50y_ versus the lowest was 4.07 (95% confidence interval (CI): 2.64, 6.25). Lower early-life SEP was associated with higher FI_50y_. Compared with participants born in the highest social class, the estimated total effect on FI_50y_ was 42.0% (95% CI: 35.5, 48.4) for participants born in the lowest class, with the proportion mediated by adult SEP being 0.45% (95% CI: 0.35, 0.55). Mediation by adult SEP was negligible for other early-life SEP classes. Findings suggest that early-life SEP is associated with frailty and that adult SEP only partially explains this association. Results highlight the importance of improving socioeconomic circumstances across the life course to reduce inequalities in midlife frailty.

## Abbreviations

CI: confidence interval
FI: frailty index
FI_50y_: frailty index at age 50 years
NDE: natural direct effect
NIE: natural indirect effect
SEP: socioeconomic position
TE: total effect

Frailty, a state of increased vulnerability resulting from age-related decline in physiological reserves (1), is associated with adverse health outcomes, including falls, hospitalizations, and premature mortality (1, 2). While there is no universal consensus regarding the operationalization of frailty (3), the 2 most common approaches define frailty as a phenotype (4) (based on 5 predefined physical frailty criteria (5)) or a frailty index (FI), based on an accumulation of health-related deficits (6, 7). Despite lack of a standard definition, there is acknowledgement that frailty presents a global challenge because of population aging (8, 9). Although the prevalence of frailty increases with age, it is not limited to older ages (10). However, most epidemiologic studies assessing predictors of frailty have focused exclusively on adults aged 65 years or more (11–13). This omission is important, because frailty reflects biological rather than chronological age (14) and is a dynamic process that may be reversible (15). However, increasing age (from 65 years onwards) is associated with a lower probability of improvement in frailty status (16). Thus, there is emerging recognition that attention to frailty in midadulthood could be pivotal in terms of identifying, managing, and preventing severe frailty at older ages (17, 18).

Reducing frailty at the population level is a desirable goal. To achieve this, a more precise understanding of predictors of frailty from midlife onwards is key to delaying its onset. A life-course approach to frailty has been discussed theoretically (19, 20) and has the potential to identify when and how to intervene at different life stages to maximize the chance of healthy population aging (19). However, to date, only a few empirical life-course studies have examined frailty. For example, a body of literature is emerging on links between early-life socioeconomic position (SEP) and frailty at older ages (13, 21–25). However, these studies have relied on relatively small sample sizes (*n* < 1,100) (13, 25) and retrospective reports of early-life SEP (21, 22). Importantly, previous studies have examined mainly older adults, and where younger adults have been evaluated (21, 23–25), the age range has been broad, with little consideration for age-related differences in associations. While associations between frailty in adulthood and mortality are well established (1), evidence suggests that frailty levels may have increased in recent generations (26). In addition, some (27) but not all (28) studies suggest that the strength of the frailty-mortality association may have weakened in more recent generations. Thus, there is utility in examining associations of frailty with both mortality and upstream factors, such as early-life SEP, in a single-aged sample from midadulthood to help clarify when in the life course these associations emerge.

Despite the burgeoning literature linking early-life SEP to frailty, only a few studies (13, 24, 25) have examined whether this association is due to life-course continuities in disadvantage. Limited evidence suggests that adult socioeconomic circumstances fully explain associations between early-life SEP and frailty at older ages (13, 24, 25). However, these studies have had several methodological limitations. For example, use of a single indicator of adult SEP, such as occupational class (13, 25), may result in mismeasurement of the mediator, potentially undermining the analysis (29). Moreover, confounders of the adult SEP-frailty relationship, such as smoking (30, 31), that are themselves influenced by early-life SEP (32) were not accounted for. To our knowledge, no study has examined these chains of associations in midlife, and almost all have operationalized frailty as a phenotype (13, 21, 23–25), with few using an FI (22). The validated FI (6) is particularly suitable for examining frailty in midadulthood because, compared with other frailty measures (including the frailty phenotype), it is more sensitive to small changes in health status (33), making it particularly suitable for examining frailty in midadulthood, a life stage in which health deficits are accumulating at a slower rate than at older ages (34).

We aimed to address several outstanding research gaps regarding the utility of measuring frailty in midlife and the links between early-life SEP and frailty. Specifically, using data from the 1958 British birth cohort, we derived an index of frailty at age 50 years (FI_50y_). To provide construct validity and establish the utility of measuring frailty in midlife, we examined associations between FI_50y_ and mortality over an 8-year follow-up period. We then examined associations between early-life SEP and FI_50y_ and investigated whether these associations were explained by adult SEP.

## METHODS

The 1958 British birth cohort includes over 17,000 participants who have been followed since their birth in England, Scotland, or Wales during a single week in March 1958 (35). Ethical approval was given, including approval at age 50 years, by the London Multi-Centre Research Ethics Committee, and participants gave informed consent at various ages. Persons who responded in midadulthood were broadly representative of the surviving cohort (36). At age 50 years, 9,789 cohort members participated; of these, 8,711 had a valid measure of FI_50y_ (see Web Figure 1, available online at https://doi.org/10.1093/aje/kwab038) and were included in the analysis. Compared with cohort members who took part at age 50 years but had insufficient information for creation of an FI (*n* = 1,078), participants included in this study had a more favorable SEP in early life and in adulthood (Web Table 1).

### Measurement of variables

#### Frailty index.

The FI was derived following guidelines outlined elsewhere (37). An included variable was required to be 1) a health-associated deficit with a prevalence that generally increases with age and 2) a factor that is not universal in the adult population by midlife (e.g., myopia was not included, but age-related sight changes (presbyopia) were included); additionally, when considered together, 3) the included variables had to cover a range of physiological systems and processes. The FI_50y_ included 34 variables (Table 1) covering 8 broad health domains (comorbidity, physical functioning, sensory functioning, mental health, cognitive function, fatigue, sleep, and general health). Most variables were dichotomized and given a score of 1 (deficit present) or 0 (deficit absent). Following the guidelines (37), cohort members (*n* = 8,711; 89.0%) were included if they had information on at least 30 deficits. For each included individual, an FI_50y_ was generated by summing the total number of deficits reported and dividing by the total number of deficits considered (the number of deficits considered varied from 30 to 34), producing a continuous score between 0 and 1.

**Table 1:** Health Deficits Used to Construct a 34-Item Index of Frailty at Age 50 Years for Participants in the 1958 British Birth Cohort (*n* = 8,711), 2008

	**Health Deficit Value** ^**a**^				**Missing Data**	
**Domain and Health Variable**	**0**		**1**		**No.**	**%**
	**No.**	**%**	**No.**	**%**		
Comorbidity						
Asthma/wheezy bronchitis^b^	7,878	90.44	833	9.56	0	0
Type 2 diabetes^b^	8,340	95.74	371	4.26	0	0
Recurrent backache, prolapsed disc, or sciatica^b^	7,221	82.90	1,490	17.10	0	0
Body pain^c^^,^^d^	7,984	91.94	700	8.06	27	0.31
High blood pressure^b^	7,395	84.89	1,316	15.11	0	0
Stomach issues^b^^,^^e^	7,987	96.86	259	3.14	465	5.34
Kidney/bladder problems^b^	8,499	97.57	212	2.43	0	0
Persistent cough/bringing up phlegm^b^	8,349	95.84	362	4.16	0	0
Sensory factors^b^						
Hearing problems^f^	7,868	90.32	843	9.68	0	0
Eyesight problems^g^	8,441	97.11	251	2.89	19	0.22
Cognitive function^h^						
Delayed recall of 10 words	6,176	71.80	2,426	28.2	109	1.25
Immediate recall of 10 words	6,853	79.14	1,806	20.9	52	0.60
No. of animals named in 1 minute	6,402	73.93	2,257	26.1	52	0.60
No. of accurately crossed out P’s and W’s in a letter grid	6,442	75.57	2,082	24.4	187	2.15
General health						
Self-rated general health^i^	1,731	19.88	455	5.23	3	0.03
Self-rated health compared with 1 year ago^j^	7,420	85.18	193	2.22	0	0
Health limiting social activities (e.g., visiting friends, relatives)^k^	8,307	95.71	372	4.29	32	0.37
Expecting one’s health to get worse^l^	6,715	94.10	510	5.90	61	0.70
Mental health						
Physical/emotional problems interfering with normal social activities with family, friends, groups, neighbors^d^^,^^m^	7,972	91.80	712	8.20	27	0.31
Feeling so down in the dumps that nothing could cheer you up^n^	8,469	97.37	229	2.63	13	0.15
Having seen a doctor or specialist or having been to a hospital because one was feeling low, depressed, or sad (and was still symptomatic)^b^	8,404	96.50	305	3.50	2	0.02
Having seen a doctor or specialist or having been to a hospital because one was feeling generally anxious or jittery (and was still symptomatic)^b^	8,564	98.32	146	1.68	1	0.01
Physical function^o^						
Moderate physical activities (e.g., pushing vacuum, moving table, bowling, playing golf)	8,241	94.70	461	5.30	9	0.10
Lifting groceries	8,257	94.95	439	5.05	15	0.17
Climbing 1 flight of stairs	8,353	96.09	340	3.91	18	0.21
Bending, kneeling, and stooping	8,025	92.22	677	7.78	9	0.10
Walking 100 yards (91.4 m)	8,365	96.14	336	3.86	10	0.11
Bathing	8,444	96.99	262	3.01	5	0.06
Fatigue^d^						
Having a lot of energy^p^	8,179	94.05	518	5.96	14	0.16
Feeling worn out^q^	7,992	91.90	704	8.10	15	0.17
Feeling full of life^r^	8,326	95.73	371	4.27	14	0.16
Feeling tired^s^	8,323	95.71	373	4.29	15	0.17
Sleep						
Amount of time usually needed to fall asleep^t^	8,152	93.76	543	6.24	16	0.18
Getting enough sleep to feel rested upon waking in the morning^d^^,^^u^	8,097	92.99	610	7.01	4	0.05

^a^ All variables were self-reported and were given a score of 1 (deficit present) or 0 (deficit absent). Deficits that included an intermediary response (e.g., “sometimes” or “a little”) were assigned a score between 0 and 1 as appropriate.

^b^ 0 = no; 1 = yes.

^c^ 0 = none, very mild, mild, or moderate; 1 = severe or very severe.

^d^ In the past month.

^e^ Stomach issues included gallstones, gastric ulcer, acid reflux, diverticulitis, hernia, tumor, and cancer.

^f^ Hearing problems included hearing loss in 1 or both ears, tinnitus, Ménière disease, age-related degeneration, and inability to hear certain noises.

^g^ Eyesight problems included diabetes-associated eye disease, glaucoma, cataract, macular degeneration, low vision, and blindness.

^h^ 0 = upper 3 quartiles; 1 = lowest quartile.

^i^ 0 = excellent (*n* = 1,731 (19.9%)); 0.25 = very good (*n* = 2,906 (33.4%)); 0.5 = good (*n* = 2,544 (29.2%)); 0.75 = fair (*n* = 1,072 (12.3%)); 1 = poor (*n* = 455 (5.2%)).

^j^ 0 = much better/the same (*n* = 7,420 (85.2%)); 0.5 = worse (*n* = 1,098 (12.6%)); 1 = much worse (*n* = 193 (2.2%)).

^k^ 0 = a good bit/some/a little/none of the time (*n* = 8,307 (95.7%)); 1 = all/most of the time (*n* = 372 (4.3%)).

^l^ 0 = don’t know/mostly false/definitely false (*n* = 6,715 (77.6%)); 0.5 = mostly true (*n* = 1,425 (16.5%)); 1 = definitely true (*n* = 510 (5.9%)).

^m^ 0 = not at all/slightly/moderately (*n* = 7,972 (91.8%)); 1 = quite a bit/extremely (*n* = 712 (8.2%)).

^n^ 0 = a good bit/some/a little/none of the time (*n* = 8,469 (97.4%)); all/most of the time (*n* = 229 (2.6%)).

^o^ 0 = limited a little/not limited; 1 = limited a lot.

^p^ 0 = all/most/some of the time (*n* = 7,159 (82.3%)); 0.5 = a little of the time (*n* = 1,021 (11.7%)); 1 = none of the time (*n* = 518 (6.0%)).

^q^ 0 = a good bit of the time/some of the time/a little of the time/none of the time (*n* = 7,992 (91.9%)); 1 = all/most of the time (*n* = 704 (8.1%)).

^r^ 0 = all/most of the time/a good bit of the time/some of the time/a little of the time (*n* = 8,326 (95.7%)); 1 = none of the time (*n* = 371 (4.3%)).

^s^ 0 = a good bit of the time/some of the time/a little of the time/none of the time (*n* = 8,323 (95.7%)); 1 = all/most of the time (*n* = 373 (4.3%)).

^t^ 0 = 1 hour or less (*n* = 8,152 (93.8%)); 1 = over 1 hour (*n* = 543 (6.2%)).

^u^ 0 = all/most/a good bit/some/a little bit of the time (*n* = 8,097 (93.0%)); 1 = none of the time (*n* = 610 (7.0%)).

#### Mortality.

Information on deaths occurring from 2008 (when cohort members were aged 50 years) to the end of 2016 (when cohort members were aged 58 years) was ascertained from a variety of sources, the majority (*n* = 198; 94.7%) through linkage to death certificates from the National Health Service Central Register (38). Information obtained from relatives or close friends during survey activities/cohort maintenance allowed identification of 11 further deaths (see Web Table 2 footnotes).

#### Early-life SEP.

Early-life SEP was identified from prospectively recorded information on father’s occupation at birth in 1958 or, if missing, at age 7 years in 1965 (*n* = 631; 7.24%). Using the Registrar General’s Social Classification groupings, 4 SEP categories were identified: professional/managerial (classes I/II), skilled nonmanual (class III nonmanual), skilled manual (class III manual), and partly skilled/unskilled manual (classes IV/V and cases where there was no male head of the household).

#### Adult SEP.

Adult SEP was considered a potential intermediary factor based on established associations with both early-life SEP (39, 40) and frailty (26, 41). It was represented by occupational class at age 42 years (or, if missing, at age 33 years (*n* = 829; 9.52%)), educational attainment by age 33 years, and household tenure at age 45 years (see Table 2 footnotes).

**Table 2:** Early-Life and Adult Socioeconomic Characteristics and Frailty Index Score at Age 50 Years in the 1958 British Birth Cohort (*n* = 8,711), 1958–2008^a^

**SEP Variable**	**Total Population**		**Women**		**Men**	
	**No.**	**%**	**No.**	**%**	**No.**	**%**
Early-life SEP^b^ (father’s occupational class)						
I/II	1,671	19.7	856	19.5	815	20.0
III nonmanual	877	10.4	439	9.98	438	10.8
III manual	4,054	47.9	2,110	48.0	1,944	47.8
IV/V	1,865	22.0	994	22.6	871	21.4
Adult occupational class^b^						
I/II	3,368	42.1	1,530	36.9	1838	47.8
III nonmanual	1,836	23.0	1,449	35.0	387	10.1
III manual	1,475	18.5	288	6.95	1,187	30.8
IV/V	1,313	16.4	876	21.1	437	11.4
Adult educational level^c^						
<O-levels	1,596	21.4	944	23.8	652	18.6
O-levels	2,548	34.1	1,503	37.9	1,045	29.8
A-levels	2,245	30.1	1,013	25.6	1,232	35.1
Degree or higher	1,083	14.5	505	12.7	578	16.5
Adult housing tenure^d^						
Owner	7,346	84.4	3,816	84.4	3,530	84.5
Renter	1,145	13.2	618	13.7	527	12.6
Other	221	2.4	88	2.0	123	2.9
Frailty index score^e^	0.07 (0.04–0.13)		0.07 (0.03–0.13)		0.07 (0.04–0.13)	

Abbreviation: SEP, socioeconomic position.

^a^ Based on observed (i.e., unimputed) data.

^b^ Early-life SEP based on father’s occupation at birth (or, if missing, at age 7 years) and adult occupational class at age 42 years (or, if missing, at age 33 years). Both were classified using the United Kingdom Register General’s classification of occupations and grouped into the following categories: professional/managerial (classes I/II), skilled nonmanual (class III nonmanual), skilled manual (class III manual), and partly/unskilled manual (classes IV/V; in early life, also included cases where there was no male head of the household).

^c^ Educational attainment based on the participant’s highest educational qualification by age 33 years. O-levels: high school qualifications, typically ascertained at age 16 years. A-levels: national qualifications, typically ascertained at age 18 years.

^d^ Housing tenure based on financial circumstances of the participant’s housing arrangements at age 45 years (or, if missing, at age 42/50 years). Tenure was grouped into 3 categories: owning a property (outright or with a mortgage), renting (or having a partial mortgage), and other (e.g., living rent-free with a relative).

^e^ Values are expressed as median (interquartile range).

#### Confounders.

**Figure 1:**
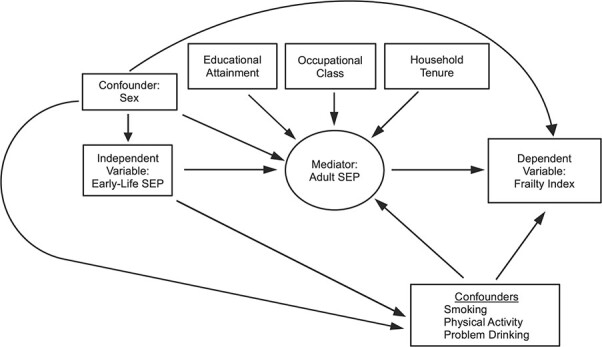
Proposed relationships between early-life socioeconomic position (SEP), adult SEP, and frailty. Boxes represent observed variables; the circle represents a latent variable. (See Methods section of the text for details.)

Confounders were identified a priori on the basis of factors associated with early-life SEP, adult SEP, and frailty. These included sex as a baseline confounder and physical activity (42, 43), smoking (30, 31), and problem alcohol drinking (30, 44) as confounders of the adult SEP-frailty relationship that are influenced by early-life SEP (32, 43, 45) (see Figure 1 and Web Appendix 1).

### Statistical analysis

Proportional hazards for mortality were visually assessed using Kaplan-Meier plots. Cox proportional hazards models were used to estimate (sex-adjusted) hazard ratios and 95% confidence intervals for associations between FI_50y_ and all-cause mortality at ages 50–58 years. Associations between FI and mortality are commonly examined using a continuous measure (46) or prespecified FI categories (0–0.1, 0.1–0.2, etc.) (2, 47, 48). In midlife, the FI is highly skewed (e.g., approximately 60% of the sample has an FI < 0.1); thus, these categorizations were not appropriate. Therefore, similar to other investigators, we divided FI_50y_ into quintiles (49). Survival time included time from completion of the age-50 survey to the date of death, censoring (last date of contact), or the end of the study period (December 2016), whichever came first. Schoenfeld residuals were checked to test the assumption of proportional hazards for FI_50y_ and sex; neither violated the assumption.

Using linear regression, we examined whether associations between early-life SEP and FI_50y_ varied by sex. There was no evidence of effect modification (*P* = 0.35); hence, results from sex-adjusted analyses are presented. For ease of interpretation, FI_50y_ was log-transformed and multiplied by 100, whereby regression coefficients can be interpreted as the symmetrical percent difference in mean values (50). Before log-transforming FI_50y_, we added 0.01 to the index, as in previous work (51), to circumvent logarithmic values of 0.

### Creation of latent classes to represent adult SEP

To account for potential measurement error introduced by using a single indicator of adult SEP, we created a latent class variable using adult occupational class, educational attainment, and household tenure, which capture different aspects of SEP (52). The best-fitting class solution was based on model fit statistics: the Akaike (53) and Bayesian (54) information criteria, with lower values indicating better fit. Class discrimination was assessed using entropy, with values of 0.6 or higher indicating good class separation (55). After identification of the optimal number of classes, participants were assigned to their most likely class, which created a categorical variable representing adult SEP that was used in subsequent analyses.

### Mediation analysis

Details on the adopted counterfactual approach are given elsewhere (56). In brief, we compared scenarios whereby the entire population was assigned a specific category for early-life SEP (i.e., classes I/II (baseline), class III nonmanual, class III manual, or classes IV/V) and adult SEP (represented by a latent variable). Our targets of estimation, the total effect (TE), natural direct effect (NDE), and natural indirect effect (NIE), were estimated for each of the 3 levels of early-life SEP as compared with the baseline (classes I/II). For each level of early-life SEP, the TE on frailty was partitioned into the effect mediated via adult SEP (NIE) and a nonmediated effect (NDE). For each level of early-life SEP as compared with baseline, the TE is the percent difference in frailty comparing 2 situations: the percent difference in mean FI_50y_ if all individuals were assigned to 1) the level of early-life SEP under consideration and 2) the baseline category of early-life SEP. For each level of early-life SEP compared with the baseline, the NDE is the percent difference in frailty comparing the situations where all individuals are assigned to early-life SEP at either 1) the level under consideration or 2) the baseline level, with adult SEP set to the value that would be observed if early-life SEP were set at its baseline level. The NIE is the difference between the relevant TE and NDE. We estimated these effects using the g-computation procedure in STATA, version 15 (StataCorp LLC, College Station, Texas) (56) (see Web Appendix 2). This procedure allowed us to estimate the effects of interest while allowing for confounders of the relationship between adult SEP and frailty that are influenced by early-life SEP.

We conducted sensitivity analysis to determine whether any single item or one of the 8 domains in the FI was particularly important for examined relationships. We systematically removed one item (or domain) at a time from the FI and examined associations between 1) FI_50y_ (minus the single item/domain) and subsequent mortality and 2) early-life SEP and FI_50y_ (minus the single item/domain).

The prevalence of missing data ranged from 2.8% (early-life SEP) to 21.0% (problem drinking at age 45 years). To minimize data loss, missing information for confounders, early-life SEP, and adult SEP was imputed using chained equations. Following guidelines (57), the imputation models included all substantive variables and the main predictors of missingness (childhood internalizing and externalizing behaviors and cognitive ability) (36). Linear regression analyses were carried out across 20 imputed data sets; overall estimates were obtained. For the g-computation procedure, a single imputation was used (bootstrapped standard errors were used to calculate 95% confidence intervals).

## RESULTS

As expected, the FI_50y_ was right-skewed, with a median value of 0.07 for both men and women, corresponding to an expression of approximately 2.4 (34 × 0.07) health-related deficits (Table 2). While 20% of the cohort had fathers in the highest occupational category (I/II) when they were born, 42% were themselves in this occupational category at age 42 years.

### Associations between early-life SEP, FI_50y_, and mortality

**Figure 2:**
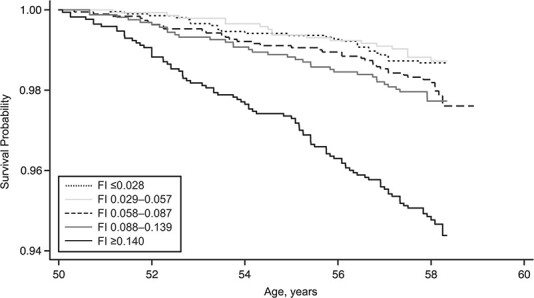
Sex-adjusted Kaplan-Meier survival probabilities according to quintile of frailty index (FI) score in the 1958 British birth cohort (*n* = 8,711; 209 deaths), 2008–2016. Higher FI scores represent higher levels of frailty.

Sex-adjusted Kaplan-Meier curves indicated that mortality generally increased progressively with increasing levels of frailty (Figure 2). For example, compared with the least frail adults, the sex-adjusted hazard ratio was 1.66 (95% CI: 1.01, 2.74) for adults in the fourth-highest quintile of frailty and 4.07 (95% CI: 2.64, 6.25) for the most frail adults (Web Table 2). Associations between FI and mortality remained stable when we systematically excluded a single item or domain from the FI. For example, per increase in frailty quintile, the sex-adjusted hazard ratio for mortality was 1.48 (95% CI: 1.32, 1.67); removing a single item from the index resulted in hazard ratios ranging from 1.40 (95% CI: 1.28, 1.54) to 1.54 (95% CI: 1.38, 1.71) when “self-reported health” and “number of animals named in 1 minute” were removed, respectively. When the domains were removed, hazard ratios ranged from 1.38 (95% CI: 1.24, 1.54) for cognitive function to 1.50 (95% CI: 1.33, 1.69) for comorbidity (Web Figure 2A).

Lower early-life SEP was associated with greater percent differences in FI_50y_. For example, each decrease (on a 4-point scale) in early-life SEP was associated with a 14.2% (95% CI: 12.1, 16.3) increase in FI. Associations between early-life SEP and FI_50y_ were broadly stable when a single item or domain was removed from the FI. For example, percent increases in FI ranged from 11.2% (95% CI: 9.48, 13.1) to 14.8% (95% CI: 12.7, 16.9) when “self-reported health” and “expect health to get worse” were removed, respectively. Associations ranged from 14.2% (95% CI: 12.1, 16.4) to 10.2% (95% CI: 7.56, 12.5) when the domains “sensory functioning” and “cognitive function” were respectively removed (Web Figure 2B).

### Latent classes representing adult SEP

We considered 2–5 latent classes. The 4-class solution was our chosen solution, because it had the smallest Akaike and Bayesian information criteria and a satisfactory entropy score (Web Table 3). The predicted probabilities for each of the 4 latent class memberships for adult occupational class, education, and tenure are shown in Web Table 4.

### Mediation analysis

The estimated TE of early-life SEP on FI_50y_ was 10.3% (95% CI: 3.0, 17.7) for participants with fathers in the class III nonmanual SEP category, 27.8% (95% CI: 22.2, 33.3) for those with fathers in the class III manual category, and 42.0% (95% CI: 35.5, 48.4) for those with fathers in the classes IV/V category when compared with participants with fathers in the classes I/II category (Table 3). When partitioned, compared with participants with fathers in classes I/II, the estimated NDEs for participants with fathers in the class III nonmanual, class III manual, and classes IV/V categories were 15.3% (95% CI: 7.9, 22.7), 32.4% (95% CI: 26.6, 38.1), and 23.0% (95% CI: 16.4, 29.7), respectively. Mediation by (the latent variable representing) adult SEP was negligible, except for participants with fathers in classes IV/V, where the proportion mediated was 0.45% (95% CI: 0.35, 0.55).

**Table 3:** Total, Natural Direct, and Natural Indirect Effects of Socioeconomic Position at Birth on Frailty Index Score at Age 50 Years^a^ and Proportion Mediated^b^ by Socioeconomic Position at Birth^c^ in the 1958 British Birth Cohort (*n* = 8,711), 1958–2008^d^

	**SEP at Birth** ^e^ ^,^ ^f^					
**Effect**	**Class III Nonmanual**		**Class III Manual**		**Classes IV/V**	
	**Mean %** **Difference**	**95% CI**	**Mean %** **Difference**	**95% CI**	**Mean %** **Difference**	**95% CI**
Total effect	10.35	3.02, 17.67	27.75	22.23, 33.27	41.96	35.48, 48.44
Natural direct effect (not via adult SEP)	15.28	7.88, 22.69	32.36	26.59, 38.14	23.03	16.36, 29.70
Natural indirect effect (via adult SEP)	−4.94	−7.77, −2.10	−4.61	−7.28, −1.95	18.93	15.49, 22.38
Proportion mediated (via adult SEP)	−0.48	−20.39, 19.43	−0.17	−0.28, −0.06	0.45	0.35, 0.55

Abbreviations: CI, confidence interval; SEP, socioeconomic position.

^a^ Mean percentage difference in frailty index score.

^b^ The proportion mediated was the natural indirect effect divided by the total effect.

^c^ Mediator: adult SEP.

^d^ The analysis adjusted for sex and for adult physical activity, smoking, and problem alcohol drinking as confounders of the adult SEP–frailty relationship that are themselves influenced by early-life SEP (see text, Figure 1, and Web Appendix 1 for details).

^e^ SEP at birth was measured as father’s occupational class at birth or, if missing, at age 7 years.

^f^ Reference group: classes I/II.

## DISCUSSION

Our study examining early-life SEP in relation to the accumulation of health-related deficits by midadulthood, carried out in a general population sample, is important for several reasons. First, we showed that by midlife, health deficits have begun to accumulate. For example, a median FI of 0.07 indicates that half the population at age 50 years had at least 2 of the considered health deficits, and similarly, one-quarter of the population had at least 4 deficits. Second, it is noteworthy that this accumulation of deficits at a relatively young age was strongly associated with mortality up to 8 years later. For example, morality hazards were 4 times higher when comparing adults with the most deficits (≥5) to those with the fewest (0–1). Third, lower SEP in early life was associated with higher levels of frailty by midadulthood, such that the estimated TE on FI_50y_ was 42% greater for participants born in the lowest SEP category as compared with the highest. Finally, compared with those born in the highest SEP category, the effect of early-life SEP on frailty for participants born in the lowest SEP category was partly explained by continuities in disadvantage into adulthood.

A major strength of this study over previous work was the examination of an age-homogenous sample. Age is strongly associated with frailty (7), and this influence could be eliminated in our study. Further strengths included examination of a large general-population sample with data collected prospectively from birth, a validated measure of frailty capturing multiple health domains, and analysis that accounted for confounders of the adult SEP–frailty relationship. We acknowledge that there is no single best indicator of SEP (52). We used father’s occupation at birth to represent early-life SEP because it is a commonly used measure, reflecting a wide range of early-life social and economic indicators, including household educational attainment, income level, and social standing. In addition, rather than using a single measure of SEP in adulthood, we constructed a latent variable capturing 3 different well-established aspects of adult SEP (52). Health deficits accumulate at a slower rate in midlife than at older ages (34), and frailty measured in younger populations might be clinically and biologically different from that measured in older populations (1). Nonetheless, our measure of frailty is particularly suited to midlife because it has demonstrated good construct validity at this life stage (58) and it provides a continuous score for the range of fitness to frailty (6), allowing detection of small differences in health as compared with other measures (33). Regarding temporality, our indicators of adult SEP were captured prior to age 50 years, when the FI was constructed. This provides some evidence supporting the temporal sequence of events, which is further bolstered by evidence from several cohort studies showing associations between adult SEP and a range of subsequent health conditions (59). However, we do acknowledge that health can also influence subsequent SEP (30). Our mediation analysis relied on several assumptions, including no unmeasured confounding and accurate parametric modeling; but required assumptions were less stringent than those for conventional alternative analyses. Finally, as with all long-term studies, attrition occurred over time. Although participants in this study had more favorable early-life and adult SEP compared with those not included, in general the sample remained broadly representative of the original cohort (36). We prevented further sample reductions due to missing data by using imputation.

Our findings that health deficits have already begun to accumulate by age 50 years and are associated with subsequent mortality agree with the literature on the accumulation of health deficits in midlife (47, 60). Although the implications of frailty in clinical practice may vary by age, we and other researchers (18, 60) have demonstrated the utility of measuring frailty earlier in the life course. Thus, our findings emphasize that measuring frailty at a particular age is meaningful in identifying persons at risk of adverse health outcomes and, because frailty is progressive, beginning with a preclinical stage, there are opportunities for early prevention (1). Furthermore, our finding that the early-life SEP–frailty and frailty–mortality associations were not largely driven by any single item or health domain suggests that the value of the FI exceeds any single item/domain.

Our results are consistent with previous studies showing that lower early-life SEP is associated with greater risk of frailty in adulthood (13, 24, 25) such that, compared with persons born in the highest SEP category, those born in the lowest category had a 42% higher FI at age 50 years. However, we found the estimated effect of early-life SEP on frailty mediated by adult SEP to be noteworthy only for the lowest (compared with the highest) SEP category at birth. Since adult SEP did not fully explain early-life SEP associations in this cohort, other explanatory pathways may be involved. Evidence suggests that early-life socioeconomic disadvantage may lead to poor adult health via biological embedding (61). For example, abnormal biological changes have been observed in adults who experienced early-life socioeconomic disadvantage in this cohort (62) and elsewhere (63), which have been proposed to lead to accelerated aging (64). In addition, pathways operating via obesity and physical activity may be relevant and warrant further research. Findings that the estimated direct effects of the 2 intermediate early-life SEP categories were larger than the corresponding estimated TEs were surprising and contrasted with previous studies in which adult SEP fully explained early-life SEP–frailty associations. Discrepancies may be due to the single-aged sample examined here as compared with the broad age ranges previously examined (24, 25) or the younger age of adults in this study compared with others (13). Notwithstanding this difference, our finding that adult SEP is an important intermediary factor through which early-life disadvantage is associated with midlife frailty agrees with other studies. Therefore, our findings, together with other evidence, suggest that interventions to improve the adult socioeconomic circumstances of persons from disadvantaged backgrounds may reduce the burden of frailty in midlife and beyond.

In conclusion, our findings have several practical and policy-relevant implications. They emphasize the value of using previously collected health data to identify persons who may be vulnerable to accelerated aging earlier in the life course. Derivations of the FI are widely used in clinical and primary-care settings in England (6, 65) to systematically identify the extent of frailty in adults aged 65 years and over. Our findings suggest that similar assessments could be valuable in midadulthood and suggest that in a primary-care setting, in addition to considering single health deficits in midlife, the accumulation of deficits is also important. Identifying adults in midlife who could benefit from early interventions might reduce the burden of frailty at older ages, improving quality of life and reducing health-care costs (18, 60). We highlight the importance of improving socioeconomic conditions over the whole life course in order to reduce health inequalities. Thus, a potential intervention focus could be on improving socioeconomic opportunities available in adulthood for those disadvantaged in childhood. Moreover, relative child poverty in the United Kingdom is projected to rise from 29.7% to 36.6% between 2018 and 2022 (66); thus, our findings underscore the importance of much-needed policies to redress socioeconomic inequalities in childhood, because they have the potential to improve health in midadult life and beyond.

## Supplementary Material

Web_Material_kwab038
