# Supplementary material for: Early-Life Socioeconomic Position and the Accumulation of Health-Related Deficits by Midlife in the 1958 British Birth Cohort Study
Source: Am J Epidemiol. 2021 Feb 17;190(8):1550–60. doi: 10.1093/aje/kwab038 (PMC8327203; doi:10.1093/aje/kwab038)
Supplement: Web_Material_kwab038 [file web_material_kwab038.pdf]

WEB MATERIAL

Early-Life Socioeconomic Position and the Accumulation of Health-Related Deficits by Midlife in the 1958 British Birth Cohort Study

Nina T. Rogers, Joanna M. Blodgett, Samuel D. Searle, Rachel Cooper, Daniel H. J. Davis, and Snehal M. Pinto Pereira

Contents:

Web Figures 1 and 2
Web Tables 1–4
Web Appendix 1
Web Appendix 2

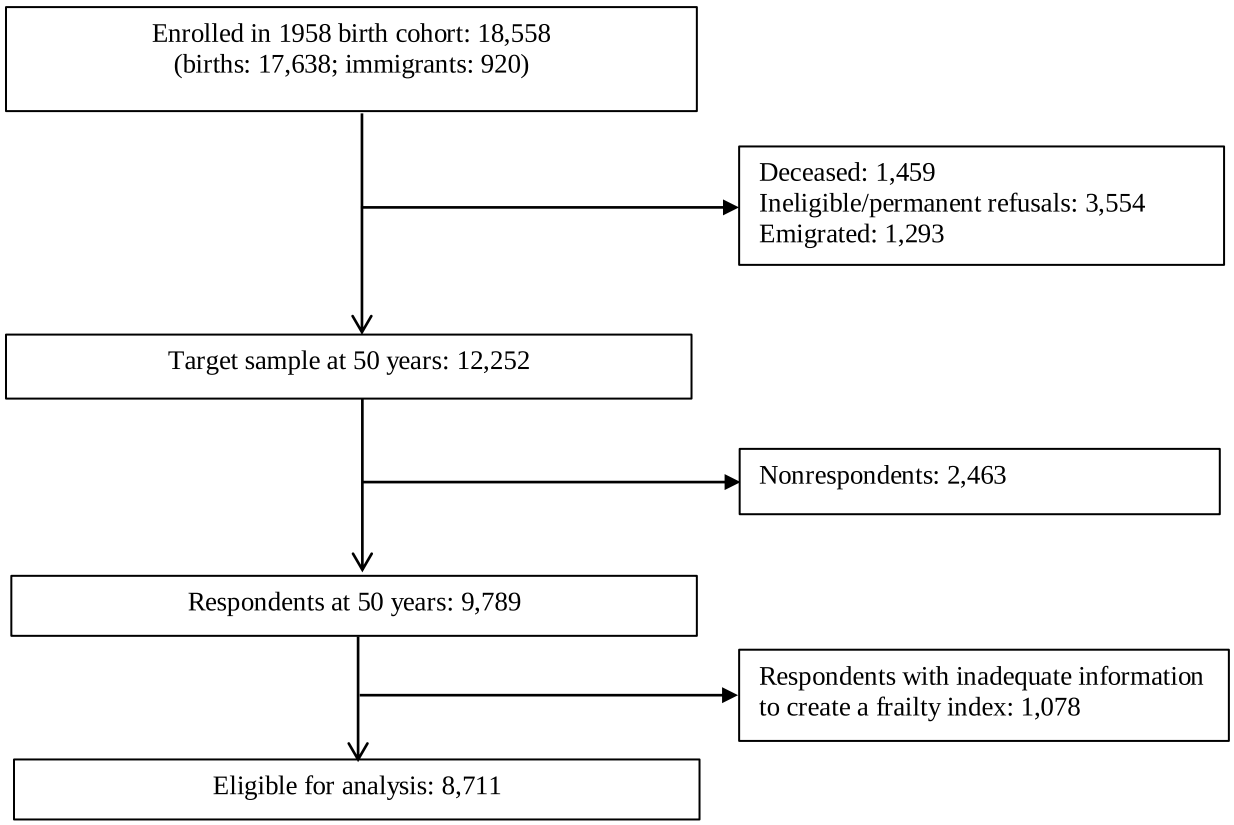

Web Figure 1. Flow diagram of participants from birth who were eligible to be included in analytical sample.

Web Figure 2A. Hazard ratios of all-cause mortality (95% confidence intervals) per increase in quintiles of specified FIs in 1958 British birth cohort participants.

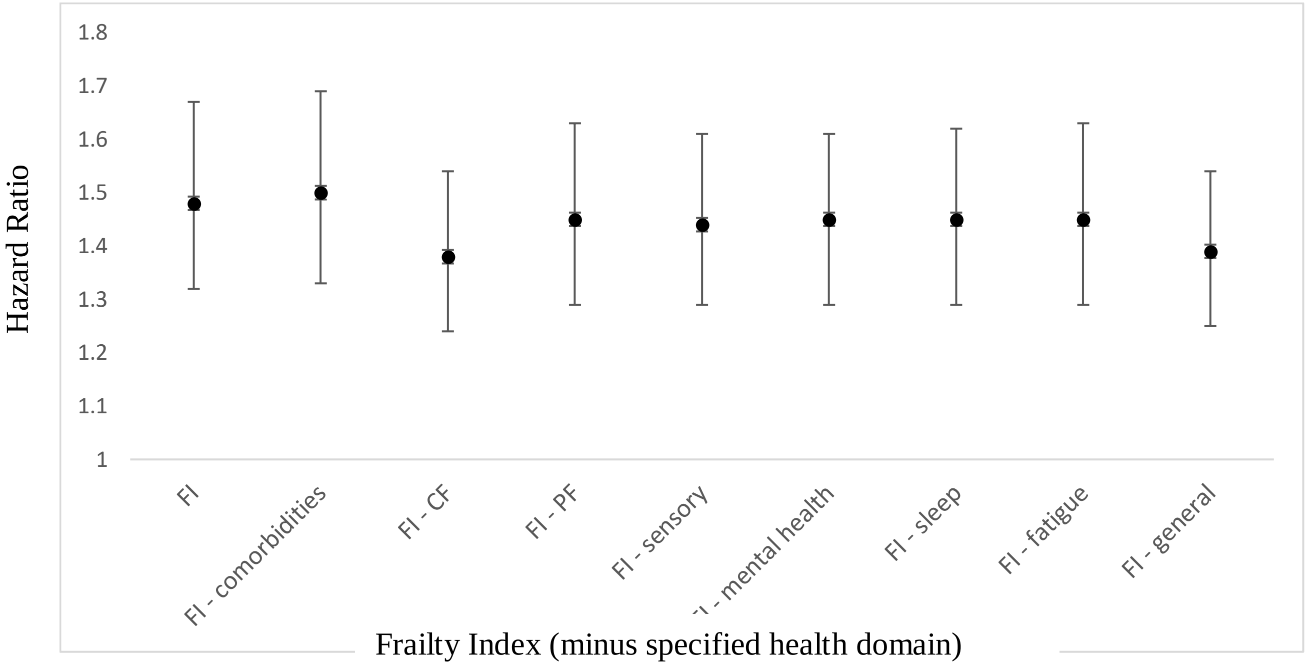

Web Figure 2B. Mean percent difference (95% confidence interval) in specified FI_{50y} per decrease in early-life SEP (on a 4-point scale).

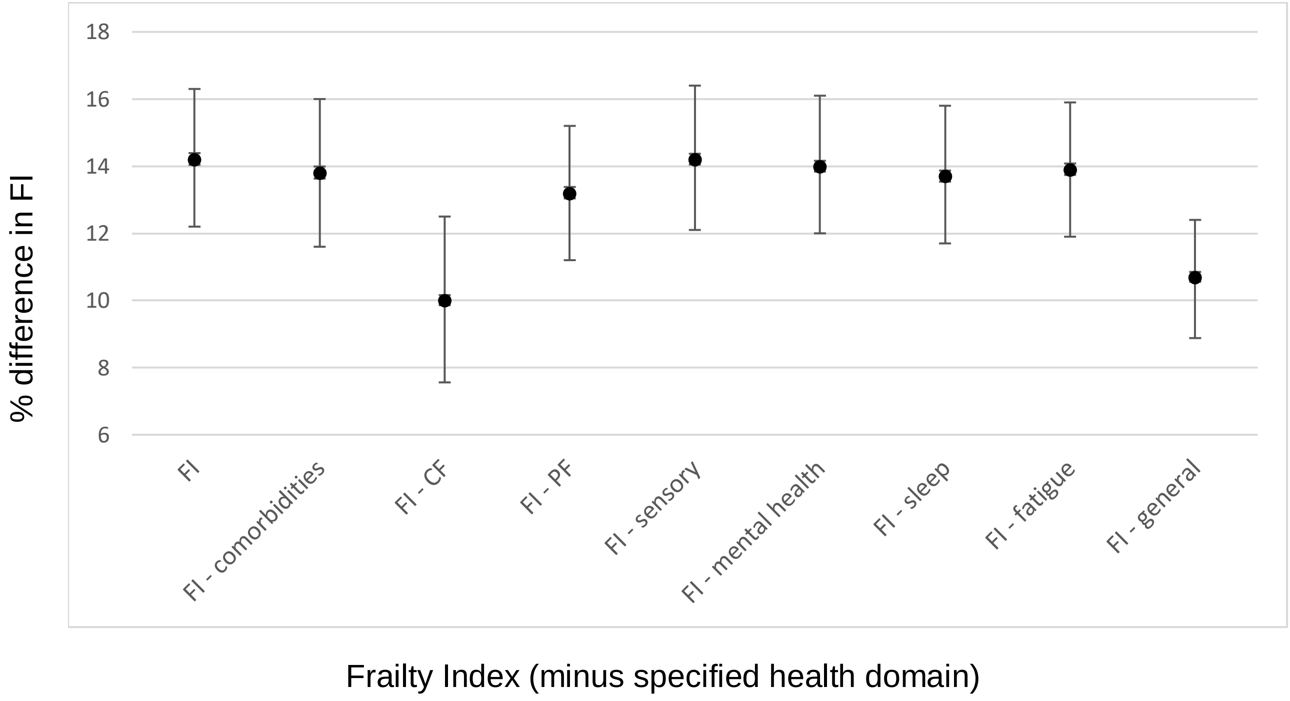

FI = 34-item FI
FI – (health domain) = 34-item FI minus specific health domain (see Table 1)
CF = cognitive function
PF= physical function

Web Table 1. Prevalence[a] of early-life socioeconomic position, and adult occupational class, educational attainment and housing tenure in the included and not included cohort at 50 years

	**Included[b] (*n* = 8,711)**	**Not Included[b] (*n* = 1,078)**
Early-life SEP[c] (father's occupational class)		
I/II	1671 (19.7)	128 (12.5)
III nonmanual	877 (10.36)	82 (7.99)
III manual	4054 (47.9)	549(53.5)
IV/V	1865 (22.0)	267(26.0)
Adult occupational class[c]		
I/II	3368 (42.1)	326 (36.3)
III nonmanual	1836 (23.0)	179(19.9)
III manual	1475 (18.5)	222 (24.7)
IV/V	1313 (16.4)	172 (19.1)
Adult education[d]		
<O-levels	1596 (21.4)	229 (28.0)
O-levels	2548 (34.1)	282 (34.5)
A-levels	2245 (30.1)	224 (27.4)
Degree or higher	1083 (14.5)	83 (10.2)
Adult housing tenure[e]		
Owner	7346 (84.4)	828 (78.8)
Renter	1145 (13.2)	193 (18.4)
Other	211 (2.42)	30 (2.85)

[a] Table based on observed (*i.e.* unimputed) data.

[b] Included: had sufficient items (≥30) to create a FI; not included: had fewer than 30 items.

[c] Early-life SEP based on father's occupation at birth (or if missing at 7 years); adult occupational class at 42 years (or if missing at 33 years). Both classified using Register General's classification of occupation and grouped into: professional/managerial (classes I and II), skilled nonmanual (class III nonmanual), skilled manual (class III manual) and partly/unskilled manual (classes IV and V; in early–life also included cases where there was no male head of household).

[d] Educational attainment based on highest educational qualification by 33 years. O-levels: high school qualifications typically ascertained at age 16 years. A-levels: National qualifications typically ascertained at age 18 years.

[e] Housing tenure based on financial circumstances of housing arrangements at 45 years (or 42/50 years if missing). Grouped into: owning property (outright or with a mortgage); rent (or part mortgage) and other (e.g. living rent-free with relative).

Web Table 2. Hazard ratios (95% confidence intervals) for quintiles of the frailty index at 50 years in relation to all-cause mortality in 1958 birth cohort participants aged 50–58 years[a] (*n* = 8,711)

Quintile of the Frailty Index[b]	No. of Participants	No. of Deaths	Death Rate (per 100 Population) at Ages 50–58 Years)	Hazard Ratio (95% CI)
1st (least frail)	2045	27	1.32	Ref
2nd	1440	18	1.25	0.94 (0.52, 1.71)
3rd	1907	38	1.99	1.48 (0.90, 2.42)
4th	1617	36	2.23	1.66 (1.01, 2.74)
5th (most frail)	1702	90	5.29	4.07 (2.64, 6.25)

[a] Models adjusted for sex. Date of death was ascertained through receipt of death certificates to the Centre for Longitudinal Studies from the National Health Service Central Register (*n* = 198) i.e. data missing for 11 individuals (for details see: National Child Development Study Deaths Dataset, 1958–2016 UK Data Service). Using survey/cohort maintenance data we determined if the deceased died between 50–55 years (*n* = 8) or 55–58 years (*n* = 3). Date of death was estimated as the mid-point between these ages.

[b] Quintiles of frailty approximately correspond to the following frailty index scores: $0 \leq FI < 0.029$ (least frail); $0.029 \leq FI < 0.058$; $0.058 \leq FI < 0.088$; $0.088 \leq FI < 0.140$; $FI \geq 0.140$ (most frail).

Web Table 3. AIC, BIC and entropy values used to determine the optimal number of latent classes to represent adult socioeconomic position

No. of Latent Classes	AIC	BIC	Entropy
2	47,310.28	47,430.50	0.682348
3	46,951.29	47,135.15	0.711840
4	*46,903.86*	*47,130.15*	*0.692061*
5	46,923.87	47,171.39	0.617084

AIC, Akaike information criterion; BIC, Bayesian information criterion.
Italic type indicates the selected model.

Web Table 4. Predicted proportions and probability of latent class membership for adult occupation, education and housing tenure

	Latent Class			
	**1**	**2**	**3**	**4**
Latent class proportion (%)	30.4	19.0	30.4	20.3
Adult occupational class				
I/II	0.911	0.485	0.114	0.057
III nonmanual	0.083	0.130	0.526	0.199
III manual	$1.29e^{-06}$	0.328	0.204	0.249
IV/V	0.006	0.057	0.155	0.494
Adult education				
<O-levels	0.017	0.139	0.0913	0.675
O-levels	0.115	0.312	0.723	0.244
A-levels	0.404	0.512	0.186	0.074
Degree or higher	0.464	0.036	0.000	0.007
Housing tenure				
Owner	0.946	0.881	.0.901	0.625
Renter	0.036	0.08	0.084	0.350
Other	0.019	0.040	0.015	0.025

Group 1: professional/ managerial occupations with high levels of education and higher than average probability of being homeowners.
Group 2: professional/managerial or skilled manual occupations with school leaving qualifications.
Group 3: predominantly skilled (nonmanual and manual) workers with school leaving qualifications, mostly owning their own home.
Group 4: predominantly from manual/unskilled occupations, low educational levels and higher than average probability of renting their home.

Results show the probability of class membership (*e.g.* individuals in latent class 1 have a 91.1% likelihood of being in adult occupational class I/II).

Web Appendix 1

G-Computation Methodology

For details of the implemented g-computation procedure, see Daniel at al. (1). In brief, we expanded our original data 1,000 times (in order to minimise Monte Carlo error in the simulations), whilst retaining an identifier of the original records (to ensure that the model parameters are estimated using the original data, rather than the expanded data). Standard errors and confidence intervals were obtained by taking 1,000 bootstrap samples (from the original dataset). The specified parametric models for simulation under different levels of early-life SEP are described in Web Appendix Table 1 below and illustrated in Figure 1. The g-formula package performs a single stochastic imputation using chained equations. In all models for imputation of missing values, predictors of missingness, as described in the main text, were included.

Web Appendix Table 1. Variable details and summary of specified parametric models

Variable (Age at Measurement)	Type of Model When Used as a Dependent Variable	Variables Specified in Parametric Model	Functional Form When Used as a Predictor
Physical activity[a] (42 years)	Ordinal logistic	Social class at birth, sex	4 categories
Smoking[b] (42 years)	Multinomial logistic	Physical activity, social class at birth, sex	3 categories
Problem drinking[c] (45 years)	Ordinal logistic	Smoking, physical activity, social class at birth, sex	4 categories
Adult SEP[d] (33–45 years)	Multinomial logistic	Problem drinking, smoking, physical activity, social class at birth, sex	4 categories
Frailty[e] (50 years)	Linear regression	Adult SEP, problem drinking, smoking, physical activity, social class at birth, sex	N/A

[a] Physical activity at 42 years was grouped into four frequency categories from ≤2–3 times per month to 4–7 times/week.
[b] Smoking status at 42 years was categorized into never, ex and current smoker.
[c] Problem drinking at 45 years was categorized into 4 groups using the AUDIT scale (2): low risk, risky or hazardous behaviour, high risk, and almost certainly dependent.
[d] Adult SEP is represented by a 4-category latent variable (see "Creation of latent classes to represent adult SEP" for details).
[e] Modelled as $\ln(FI_{50y}) \times 100$ (see "Statistical analysis" for details).

Web Appendix 2

STATA Code and Output From the G-Formula Command

Variables used in the command line (see Methods for details):

lnfrail100: ln(frailty index)*100

scbirthx: social class at birth

predclass: adult social class (represented by a 4-category latent variable)

n622: sex

actty41: physical activity at 42 years

audit_completecase_4c: problem drinking at 45 years

smok42_1: smoking at 42 years

zint711: internalising behaviours at 7 years (or if missing at 11 years), a predictor of missingness

zext711: externalising behaviours at 7 years (or if missing at 11 years), a predictor of missingness

zmath711: mathematical ability at 7 years (or if missing at 11 years), a predictor of missingness

---

STATA code

```
gformula lnfrail100 scbirthx predclass n622 actty41 audit_completecase_4c smok42_1
zint711 /*
*/ zext711 zmath711, mediation outcome(lnfrail100) exposure(scbirthx)
mediator(predclass) /*
*/ base_confs(n622) post_confs(actty41 smok42_1 audit_completecase_4c)
baseline(scbirthx: 1) oce /*
*/ commands(lnfrail100: regress, predclass: mlogit, actty41: ologit, smok42_1:
mlogit, audit_completecase_4c: ologit ) /*
*/ equations(lnfrail100: i.predclass i.audit_completecase_4c  i.smok42_1 i.actty41
i.scbirthx i.n622, /*
*/ predclass: i.audit_completecase_4c i.smok42_1 i.actty41 i.scbirthx i.n622,
audit_completecase_4c: i.actty41 i.smok42_1 i.scbirthx i.n622, smok42_1: i.actty41
i.scbirthx i.n622, /*
*/ actty41: i.scbirthx i.n622) /*
*/ seed(81) impute(scbirthx predclass actty41 audit_completecase_4c smok42_1
zint711 zext711 zmath711) /*
*/ imp_cmd(scbirthx:ologit, predclass:mlogit, actty41: ologit,
audit_completecase_4c: ologit, smok42_1: mlogit, zint711: regress, zext711:
regress, zmath711: regress)/*
*/ imp_eq(scbirthx: i.predclass i.actty41 i.audit_completecase_4c i.smok42_1
zint711 zext711 zmath711 lnfrail100 i.n622, /*
*/ predclass : i.scbirthx i.actty41 i.audit_completecase_4c i.smok42_1 zint711
zext711 zmath711 lnfrail100 i.n622, actty41: i.predclass i.scbirthx
i.audit_completecase_4c i.smok42_1 zint711 zext711 zmath711 lnfrail100 i.n622, /*
*/ audit_completecase_4c: i.actty41 i.predclass i.scbirthx i.smok42_1 zint711
zext711 zmath711 lnfrail100 i.n622, smok42_1: i.audit_completecase_4c i.actty41
i.predclass i.scbirthx zint711 zext711 zmath711 lnfrail100 i.n622, /*
```

```
*/ zint711: i.smok42_1 i.audit_completecase_4c i.actty41 i.predclass i.scbirthx
zext711 zmath711 lnfrail100 i.n622, zext711: zint711 i.smok42_1
i.audit_completecase_4c i.actty41 i.predclass i.scbirthx zmath711 lnfrail100
i.n622,/*
*/ zmath711: zext711 zint711 i.smok42_1 i.audit_completecase_4c i.actty41
i.predclass i.scbirthx lnfrail100 i.n622) imp_cycles(10) simulations(1000)
samples(1000)

-----------------------------------------------------------------------------------
STATA output
-----------------------------------------------------------------------------------

G-computation procedure using Monte Carlo simulation: mediation

[Please note that neither the abbreviation of variable names nor the use of
variable lists (such as x1-x3 to denote x1 x2 x3) is supported by this command.
If you have used abbreviations or lists, the command may fail to run or the
results below may not be correct.]


   Outcome variable: lnfrail100
   Exposure variable(s): scbirthx
   Mediator variable(s): predclass
   Size of MC sample: 1000
   No. of bootstrap samples: 1000


   A summary of the specified parametric models:

   (for simulation under different interventions)

      Variable | Command | Prediction equation
   ------------+---------+-----------------------------------------------------
       actty41 | ologit  | i.scbirthx i.n622
      smok42_1 | mlogit  | i.actty41 i.scbirthx i.n622
   audit_co~4c | ologit  | i.actty41 i.smok42_1 i.scbirthx i.n622
     predclass | mlogit  | i.audit_completecase_4c i.smok42_1 i.actty41
               |         | i.scbirthx i.n622
    lnfrail100 | regress | i.predclass i.audit_completecase_4c i.smok42_1
               |         | i.actty41 i.scbirthx i.n622
   ---------------------------------------------------------------------------



   A summary of the specified parametric models:

   (for imputation of missing values)

      Variable | Command | Prediction equation
   ------------+---------+-----------------------------------------------------
      scbirthx | ologit  | i.predclass i.actty41 i.audit_completecase_4c
               |         | i.smok42_1 zint711 zext711 zmath711 lnfrail100 i.n622
     predclass | mlogit  | i.scbirthx i.actty41 i.audit_completecase_4c
               |         | i.smok42_1 zint711 zext711 zmath711 lnfrail100 i.n622
       actty41 | ologit  | i.predclass i.scbirthx i.audit_completecase_4c
               |         | i.smok42_1 zint711 zext711 zmath711 lnfrail100 i.n622
   audit_co~4c | ologit  | i.actty41 i.predclass i.scbirthx i.smok42_1 zint711
               |         | zext711 zmath711 lnfrail100 i.n622
      smok42_1 | mlogit  | i.audit_completecase_4c i.actty41 i.predclass
               |         | i.scbirthx zint711 zext711 zmath711 lnfrail100 i.n622
       zint711 | regress | i.smok42_1 i.audit_completecase_4c i.actty41
               |         | i.predclass i.scbirthx zext711 zmath711 lnfrail100
               |         | i.n622
```

```
      zext711 | regress | zint711 i.smok42_1 i.audit_completecase_4c i.actty41
              |         | i.predclass i.scbirthx zmath711 lnfrail100 i.n622
     zmath711 | regress | zext711 zint711 i.smok42_1 i.audit_completecase_4c
              |         | i.actty41 i.predclass i.scbirthx lnfrail100 i.n622
   ------------------------------------------------------------------------------


   No. of subjects = 8711

|--------------------------PROGRESS--------------------------|
   Performing the imputation step:
|------------------------------------------------------------|

                                            |-PROGRESS-|
   Preparing dataset for MC simulations:    |----------|

                                            |-PROGRESS-|
   Fitting parametric models and simulating:  |----------|

                                            |-PROGRESS-|
   Estimating direct/indirect effects:      |----------|


   Bootstrapping:
(running gformula_ on estimation sample)

Bootstrap replications (1000)
----+--- 1 ---+--- 2 ---+--- 3 ---+--- 4 ---+--- 5
..................................................    50
..................................................   100
..................................................   150
..................................................   200
..................................................   250
..................................................   300
..................................................   350
..................................................   400
..................................................   450
..................................................   500
..................................................   550
..................................................   600
..................................................   650
..................................................   700
..................................................   750
..................................................   800
..................................................   850
..................................................   900
..................................................   950
..................................................  1000

G-computation formula estimates of the total causal effect and the natural
direct/indirect effects

    Note: The total causal effect (TCE(k)), comparing level k
          of the exposure against the baseline, is a comparison
          between the mean potential outcome if, contrary to fact,
          all subjects were exposed at level k, and the mean
          potential outcome if all subjects received the baseline
          level of exposure. Writing X for the exposure, M for the
          mediator(s), Y for the outcome, and 0 for the baseline:

                  TCE(k)=E[Y{X=k,M(X=k)}]-E[Y{X=0,M(X=0)}]

          The natural direct effect (NDE(k)) is a comparison between the
```

mean of two potential outcomes. The first is the potential outcome if, contrary to fact, all subjects received exposure k, and subjects' mediator(s) were set to their potential value(s) under baseline exposure. The second is the potential outcome if, contrary to fact, all subjects experienced the baseline exposure. That is:

NDE(k)=E[Y{X=k,M(X=0)}]-E[Y{X=0,M(X=0)}]

The natural indirect effect (NIE(k)) is the difference between the TCE(k) and the NDE(k). That is:

NIE(k)=TCE(k)-NDE(k)=E[Y{X=k,M(X=k)}]-E[Y{X=k,M(X=0)}]

The proportion mediated (PM(k)) is the NIE(k) divided by the TCE(k).

Baseline value(s):
scbirthx=1

	G-Computation Estimate(MD)	Bootstrap SE	z	P >|z|	Normal-Based [95% CI]	
TCE(2)	10.34617	3.735372	2.77	0.006	3.024972	17.66736
TCE(3)	27.75014	2.815832	9.86	0.000	22.23121	33.26907
TCE(4)	41.96055	3.305779	12.69	0.000	35.48134	48.43976
NDE(2)	15.28394	3.776229	4.05	0.000	7.882666	22.68521
NDE(3)	32.36439	2.945003	10.99	0.000	26.59229	38.13649
NDE(4)	23.02742	3.403921	6.76	0.000	16.35586	29.69898
NIE(2)	-4.937773	1.447358	-3.41	0.001	-7.774543	- 2.101002
NIE(3)	-4.614249	1.360941	-3.39	0.001	-7.281644	- 1.946853
NIE(4)	18.93313	1.757518	10.77	0.000	15.48846	22.3778
PM(2)	-.4772562	10.15835	-.05	0.963	-20.38726	19.43274
PM(3)	-.1662784	.0560595	-2.97	0.003	-.276153	- .0564038
PM(4)	.4512126	.049691	9.08	0.000	.35382	.5486052

Web References

1. Daniel RM, De Stavola BL, Cousens SN. Gformula: estimating causal effects in the presence of time-varying confounding or mediation using the g-computation formula. *Stata J*. 2011;11(4):479–517.

2. Babor TF, Higgins-Biddle JC, Saunders JB, et al. *AUDIT: The Alcohol Use Disorders Identification Test. Guidelines for Use in Primary Health Care*. 2nd ed. Geneva, Switzerland: World Health Organization; 2001. https://apps.who.int/iris/handle/10665/67205. Accessed February 14, 2020.
